# Identification of a Mutation in the Novel Compound Heterozygous CFTR in a Chinese Family with Cystic Fibrosis

**DOI:** 10.1155/2020/6507583

**Published:** 2020-05-07

**Authors:** Hongxia Shao, Jingna Hua, Qi Wu, Xiaoge Li, Ming Zhang, Herong Wang, Junping Wu, Long Xu, Yi Xie, Li Li, Huaiyong Chen

**Affiliations:** ^1^Department of Respiratory Medicine, Haihe Hospital, Tianjin University, Tianjin 300350, China; ^2^Tianjin Institute of Respiratory Diseases, Tianjin 300350, China; ^3^Tianjin Key Laboratory of Lung Regenerative Medicine, Tianjin 300350, China; ^4^Tianjin Jinnan Xiaozhan Hospital, Tianjin 300353, China; ^5^Department of Medical Ultrasonics, Haihe Hospital, Tianjin University, Tianjin 300350, China; ^6^Department of Tuberculosis Medicine, Haihe Hospital, Tianjin University, Tianjin 300350, China; ^7^Department of Science and Education, Haihe Hospital, Tianjin University, Tianjin 300350, China

## Abstract

Cystic fibrosis (CF) is one of the most common autosomal recessive disorders among Caucasians of Northern European descent but is uncommon in the Chinese population. *Objectives*. To elucidate the mutation in the novel compound heterozygous CFTR causing CF in Chinese family. *Materials and Methods*. Clinical samples were obtained from a Chinese family, the brother and sister with recurrent airway infections, hypoxemia and obstructive ventilatory impairment, sinusitis, clubbed fingers, salty sweat, and nasal polyposis. We performed whole-exome sequencing on the family and validated all potential variants by Sanger sequencing. *Results*. Next-generation sequencing showed a novel compound heterozygous CFTR mutation (c.400 A > G p.Arg134Gly and c.3484 C > T p.Arg1162^*∗*^) which resulted in CF in the family. *Conclusions*. As this mutation is consistent with the observed clinical manifestations of CF and no other mutations were detected after scanning the gene sequence, we suggest that their CF phenotypes are caused by the compound heterozygous mutation, c.400 A > G p.Arg134Gly and c.3484 C > T p.Arg1162^*∗*^. As c.400 A > G is not currently listed in the Cystic Fibrosis Mutation Database, this information, regarding the CF-causing mutations in two Chinese patients, is of interest.

## 1. Introduction

Cystic fibrosis (CF) is one of the most common autosomal recessive disorders among Caucasians of Northern European descent, but it is uncommon in the Chinese population. It is characterized by nasal polyposis, chronic obstructive pulmonary disease, and congenital bilateral absence of the vas deferens (CBAVD). Cystic fibrosis is diagnosed by clinical symptoms, laboratory tests including immunoreactive trypsinogen (IRT) testing, and sweat chlorometry [1]. Of course, the diagnosis is more certain if the CFTR mutation is present. CF is an autosomal recessive genetic disease associated with a 250,000 base-pair region on chromosome 7q encoding cystic fibrosis transmembrane conductance regulator (CFTR). CFTR contains 1,480 amino acids and is located in the upper plasma membrane of the epithelial cells in the airway and digestive and reproductive tracts. To date, about 2000 CFTR mutations have been identified (CFGAC; http://www.genet.sickkids.on.ca/), but only hundreds of them have been clearly shown to contribute to CF [2].

Approximately 50% of Caucasian CF patients are homozygous for the ΔF508 mutation, which results in a complete loss of CFTR function and the classic, severe manifestations of the disease. About 40% of CF patients have ΔF508 on one chromosome and another less common mutation on the other chromosome. The remaining ∼10% of patients have two rare mutations [3, 4].

In European and American countries, CF is inherited as recessive mutations, and both are homozygous. However, Professor Xilun Tian's team at Peking Union Medical College Hospital reported that the CFTR mutations associated with CF in Chinese population are very different from those in western patients; the common CFTR mutation group in Europe and America was used [5]. Therefore, it is difficult to find CF patients in China using established mutation screening platforms. Even in the west, doctors are less inclined to diagnose CF in patients of Chinese origin. Heterozygotes account for a large proportion of the CF patients diagnosed in China. Here, we report a brother and sister with CF who were tested for heterozygous mutations. The results showed that they have the same compound heterozygous mutation (c.400 A > G p.Arg134Gly and c.3484 C > T p.Arg1162^*∗*^) in CFTR, with typical clinical characteristics, such as recurrent airway infections, hypoxemia and obstructive ventilatory impairment, sinusitis, clubbed fingers, salty sweat, and nasal polyposis.

In this report, we elucidated the causative role of this novel compound heterozygous mutation in the pathogenesis of a severe form of CF along with a review of previously reported cases.

## 2. Materials and Methods

### 2.1. Ethical Compliance

This study was approved by the Research Ethics Committee of Haihe Hospital, Tianjin University, and all experiments were performed in accordance with approved guidelines.

### 2.2. Participants

**Figure 1:**
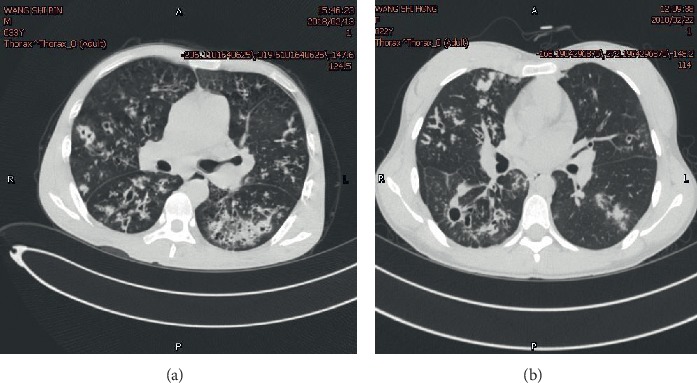
A is the image of chest CT from the male and B is from the female. Both of the images show sacculated bronchiectasis and patchy opacity in bilateral lung field. (a) The brother's chest CT. (b) The sister's chest CT.

We reported a pair of brother and sister from one Chinese family. The brother is a 33-year-old male patient with intermittent cough and sputum coughing for more than 20 years, who was admitted to our hospital in January 2018 for a fever and wheezing for more than one month. He had a history of nasal polyps for more than 10 years and had surgical treatment. The patient coughed up yellow phlegm recurrently since he was young, and it would become worse when catching a cold. He was diagnosed with bronchiectasis before. One month prior to admission, his cough became worse, and the amount of phlegm increased after catching a cold. The highest temperature was 39°C, which was accompanied by chills and heavy breathing after activity. There were moist rales audible in both lungs. Finger clubbing was also present. His white blood cell count was 11.41 × 10^9^/L, with 89.5% N. Blood gas analysis showed an oxygen partial pressure of 58.1 mmHg, indicating respiratory failure. Chest CT (Figure 1(a)) showed bilateral bronchiectasis accompanying with infection. CT of the sinuses suggested paranasal sinusitis. Echocardiography indicated tricuspid regurgitation, and his pulmonary artery systolic pressure was estimated at 42 mmHg. Bronchoscopy showed large amounts of purulent yellow secretions in both bronchi, and alveolar lavage fluid was aspirated and retained for examination. The bronchoalveolar lavage fluid was cultured and yielded *Pseudomonas aeruginosa*. Lung function testing showed FEV1, 24.3% pred and FEV1/FVC, 60.31%. After anti-infection treatment, the patient's temperature was normal, his cough was improved, sputum production was decreased, and there was no wheezing.

The younger sister, who was 22 years old, was hospitalized due to cough and sputum coughing for 3 years and aggravation for 1 week. She also had a history of nasal polyp surgery. Laboratory results showed the following: WBC 12.47 × 10^9^/L, with N 76.9%. Chest CT (Figure 1(b)) showed bilateral bronchiectasis with infection, and CT of the sinuses suggested paranasal sinusitis. She also underwent a bronchoscopy, and the *Pseudomonas aeruginosa* was found in cultured Bronchoalveolar Lavage Fluid.

A sweat test was performed on both patients. For the brother, the chloride concentrations on the left and right upper limbs were 180 mmol/L and 162 mmol/L, respectively. For the sister, the chloride concentrations on the left and right upper limbs were 165 mmol/L and 147 mmol/L, respectively. The results were significantly higher than normal (<60 mmol/L is normal). The male patient was found to have blocked vasa deferentia but normal sperm activity, and via IVF (in vitro fertilization), he fathered two test-tube babies who were healthy. The female was unmarried. Their parents are not consanguineous marriage. Neither of them had a history of chronic respiratory disease besides their mother having breast cancer.

### 2.3. Sanger Sequencing

Genomic DNA was extracted from the peripheral blood of both patients according to the manufacturer's protocol (Reference sequence GRCh37/hg19; WuXi NextCODE, Shanghai, China).

Whole-exome sequencing was performed for both patients, and all exons were captured by the Agilent SureSelect Human All Exon V5 kit on an Illumina Cluster system with SBS enrichment. The average sequencing depth of the target region was≥90×, and the sequencing depth for 95% of the target sequence was greater than 20×. Base recognition was performed on all sequenced fragments. This test was established and verified by Mingma Biotechnology (CLIA laboratory ID: 99D2064856, no. 288, Fute Middle Road, Waigaoqiao Free Trade Zone, Pudong New Area, Shanghai). Sentieon software was used in the secondary analysis to analyze the sequencing data. Sentieon BWA was compared with the UCSC hg19 reference genome. The WuXi Next CODE development process was used to annotate variants. In addition, the sequencing depth and variation prediction for each base were obtained from the genomic sequencing data. Variant effect predictor was used for annotation. Three major databases containing known or suspected pathogenic variants, including ClinVar, OMIM, and HGMD, were used to screen for known pathogenic variants, and multiple tools were used to predict the function of missense variants and annotate noncoding regulatory sequences.

## 3. Results

**Figure 2:**
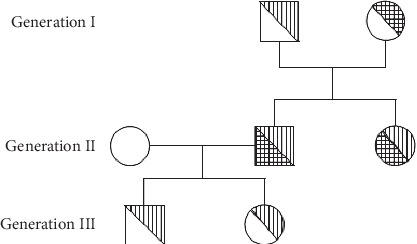
Pedigree of the CFTR gene in three generations of a family with cystic fibrosis (lined symbols in generation II). The male proband had a compound heterozygous mutation (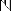 male c.400 A > G heterozygous carrier; 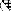 Female c.3484 C > T heterozygous carrier; 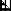
Male c.400 A > G and c.3484 C > T compound heterozygous; 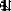 Female c.400 A > G and c.3484 C > T compound heterozygous).

**Figure 3:**
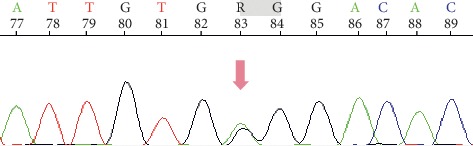
The c.400 A > G (p.Arg134Gly) variant was inherited from their father.

**Figure 4:**
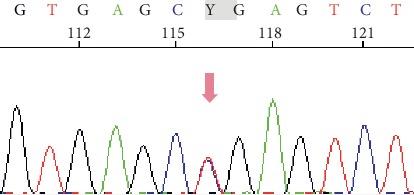
The c.3484 C > T (p.Arg1162^*∗*^) variant was inherited from their mother.

With the elder brother as the proband, we conducted genetic testing on his father, mother, sister, son, and daughter. The results are shown in Figures 2–4.

The genomic DNA sequencing indicated that the brother and sister had the same mutations in the CFTR gene. Sanger sequencing confirmed that they carried the same compound heterozygous mutation (c.400 A > G and c.3484 C > T) in CFTR. Sequencing of the parental DNA showed that the c.400 A > G mutation was inherited from the father, and the c.3484 C > T mutation was inherited from the mother.

## 4. Discussion

CF is an autosomal recessive disorder resulting from mutations in the CFTR gene that resulted in deficient or dysfunctional CFTR protein. Mutations in CFTR cause a multisystem disease, and mean survival of patients was about 40 years [6]. CFTR encodes the CFTR protein, which is mainly located in the membrane of epithelial cells. It functions as a chloride and bicarbonate channel [7] mostly in the upper and lower airways, intestine, pancreas, liver ducts, and other mucous secretion organs [8]. The degree of CFTR deficiency determines the severity of the disease. Mutations in the CFTR gene result in elevated chloride concentrations in sweat, which is an important diagnostic criterion. Smith [9] reported that at least 97% of males with CF have abnormal vasa deferentia, resulting in infertility, although sperm can be retrieved from 90% of patients using in IVF. The male patient in our study utilized IVF to produce a set of twins. The two patients we described here have nasosinusitis, bronchiectasis, high levels of chloride in sweat, and CFTR gene mutations, and the male patient is infertile due to a vas deferens abnormality; therefore, CF was diagnosed.

Generally, it was believed that patients must be homozygous for a CFTR mutation to develop CF, because carriers with a single CFTR mutation have approximately 50% of the normal CFTR function, which is enough to maintain their health [10]. However, first reports for CF in compound heterozygous patients date back to 1991 [11], only two years after the initial discovery of the CF-causing gene. In our report, the DNA sequencing revealed a previously unreported compound heterozygous mutation in the CFTR gene. Whole-exome sequencing was performed on other family members, which showed that both parents are disease-free, heterozygous CFTR gene mutation carriers. The two CF patients have the same compound heterozygous mutation in the CFTR gene (c.400 A > G p.Arg134Gly and c.3484 C > T p.Arg1162^*∗*^). One mutation (c.400 A > G p.Arg134Gly) was inherited from the father, and the other (c.3484 C > T p.Arg1162^*∗*^) was inherited from the mother. The third generation is a set of fraternal twins who have one heterozygous CFTR gene mutation (c.400 A > G p.Arg134Gly) and are disease-free.

The CFTR gene is located in chromosomal region 7q31.2 and is composed of 27 exons [11], and encodes a protein of 1,480 amino acids [12]. More than 2,000 CFTR mutations have been recorded, but only hundreds of them have been clearly shown to contribute to CF [2]. CFTR mutations are divided into six classes according to function [13]: (I) protein production, (II) protein processing, (III) protein regulation, (IV) protein conduction, (V) the amount of functional CFTR protein, and (VI) the duration of the protein on the plasma membrane [14, 15]. The phenotype of patients with class I–III mutation is more severe than that of patients with class IV–VI mutations. This is because CFTR proteins with class IV, V, and VI mutations retain some residual function, whereas CFTR proteins with class I–III mutations do not [1].

We screened all exons of CFTR and detected two mutations in the family, and one mutation, c.3484 C > T p.Arg1162^*∗*^, was previously reported. This mutation is located in exon 22 and is a C ⟶ T substitution. It is a nonsense mutation that changes the CGA codon at position 1162, which encodes arginine to a UGA stop codon (R1162^*∗*^) [16]. The other mutation of the CFTR gene (c.400 A > G p.Arg134Gly) was not previously reported to cause CF. This mutation is located at position 400 and leads to an A ⟶ G substitution (p.134). The specific effect of this mutation is unclear.

CF caused by compound heterozygous mutations were reported. Terlizzi et al. [17] investigated the relationship between CFTR genotype and phenotype (clinical manifestations or severity) and showed that clinical manifestations are caused by different mutations and that compound heterozygous mutations can cause CF. Yang et al. [18] reported that compound heterozygous mutations in the CFTR gene cause CBAVD in Chinese populations. The clinical manifestations of CF vary depending on the CFTR mutations. The c.3484 C > T variant is located in exon 22 and stops DNA transcription. It is a nonsense mutation leading to a defect in protein production, which is categorized as a class I mutation and results in a relatively severe clinical phenotype. The R1162X mutation is rare, and the class I mortality rate is about 20% [19]. Mohseni et al. [20] reported that the CFTR mutation (c.3484 C > T p.Arg1162^*∗*^) detected in our study resulted in CF when combined with a previously unreported CFTR mutation (c.3119 T > A p.L1040H). The R1162X mutation carrier in our study is healthy, without any respiratory issues or other CF manifestations. The same is true for the p.Arg134Gly carriers. This suggests that this novel, compound heterozygous mutation causes CF and results in CBAVD.

CF is common among Caucasians but is rare in Chinese populations. Until now, only dozens of CF patients have been reported in Chinese populations. However, the numbers are increasing because of genetic testing [5, 21–23]. The p.F508del mutation is present in 70% of Caucasian CF populations, which is in contrast to its rare frequency in Chinese CF patients, as most CF-causing alleles found in Chinese are rare or absent in other races, and their functional consequences have not yet been analyzed [23, 24]. This difference in the variant spectrum of CFTR mutations between Caucasians in western countries and Chinese in Asian countries could cause different symptoms in these populations. The atypical symptoms and low awareness of the disease in China make it difficult to diagnose CF. Therefore, it is necessary to increase awareness of CF in China and to establish a CFTR mutation database for the Asian population.

In conclusion, we found a novel heterozygous mutation in a Chinese family. One of the detected CFTR mutations alone, either c.3484 C > T p.Arg1162^*∗*^ heterozygous carrier or c.400 A > G p.Arg134Gly heterozygous carrier, cannot induce CF. The effect of the c.400 A > G p.Arg134Gly variation is unknown. Although these gene loci appear to be far away from one another, functional supplementation may occur. The study results suggest that the p.Arg134Gly variation may supplement the function of the R1162X heterozygous mutation to promote the development of CF. The variant spectrum in the Chinese population is different from that of the Western population; thus, a CFTR mutation database for the Asian population is greatly needed.

## Data Availability

No data were used to support this study.
